# Association between muscle quality index and pulmonary function in post-COVID-19 subjects

**DOI:** 10.1186/s12890-023-02745-5

**Published:** 2023-11-15

**Authors:** Dulce González-Islas, Robinson Robles-Hernández, Laura Flores-Cisneros, Arturo Orea-Tejeda, Susana Galicia-Amor, Nadia Hernández-López, Mariana I. Valdés-Moreno, Rocío Sánchez-Santillán, Juan Carlos García-Hernández, Armando Castorena-Maldonado

**Affiliations:** 1 Heart Failure and Respiratory Distress Clinic at the Instituto Nacional de Enfermedades Respiratorias “Ismael Cosío Villegas”, Calzada de Tlalpan 4502 Col Sec XVI CP 14080 Del Tlalpan, Mexico City, Mexico; 2 Department of Research in Tobacco Smoking and COPD at the Instituto Nacional de Enfermedades Respiratorias “Ismael Cosío Villegas”, Mexico City, Mexico; 3 Department of Epidemiological Information Analysis at Dirección General de Epidemiología, Secretaría de Salud, Gobierno de México, Mexico City, 01480 Mexico; 4 Pulmonary Rehabilitation Department at the Instituto Nacional de Enfermedades Respiratorias “Ismael Cosío Villegas”, Mexico City, Mexico; 5 Licenciatura en Nutriología Facultad de Estudios Superiores Zaragoza Universidad Nacional Autónoma de México, C.P. 09230 Mexico City, Mexico; 6 Direction for Medical Care in Pneumology at the Instituto Nacional de Enfermedades Respiratorias “Ismael Cosío Villegas”|, Mexico City, Mexico

**Keywords:** Pulmonary function, Muscle quality index, DLCO, Respiratory muscle strength

## Abstract

**Background:**

The SARS-CoV2 pandemic impacted many critically ill patients, causing sequelae, affecting lung function, and involving the musculoskeletal system. We evaluated the association between lung function and muscle quality index in severely ill post-COVID-19 patients.

**Methods:**

A cross-sectional study was conducted on a post-COVID-19 cohort at a third-level center. The study included patients who had experienced severe-to-critical COVID-19. Anthropometric measurements, such as body mass index (BMI) and handgrip strength, were obtained to calculate the muscle quality index (MQI). Additionally, spirometry, measurements of expiratory and inspiratory pressure, and an assessment of DLCO in the lungs were performed. The MQI was categorized into two groups: low-MQI (below the 50th percentile) and high-MQI (above the 50th percentile), based on sex. Group differences were analyzed, and a multivariate linear regression analysis was performed to assess the association between respiratory function and MQI.

**Results:**

Among the 748 patients analyzed, 61.96% required mechanical ventilation, and the median hospital stay was 17 days. In patients with a low MQI, it was observed that both mechanical respiratory function and DLCO were lower. The multivariate analysis revealed significantly lower findings in mechanical respiratory function among patients with a low MQI.

**Conclusion:**

The Low-MQI is an independent predictor associated with pulmonary function parameters in subjects with Post-COVID-19 syndrome.

## Background

Post-COVID-19 syndrome is characterized by the development of signs and symptoms during or after an infection caused by severe acute respiratory syndrome coronavirus-2 (SARS-CoV-2), with symptoms persisting for more than 12 weeks and not attributable to alternative diagnoses [1]. A longitudinal cohort study revealed that 68% of subjects experienced symptoms at 6 months, and 49% experienced symptoms at the 1-year mark following the initial acute viral infection caused by SARS-CoV-2 [2].

Additionally, the severity of COVID-19 infections is associated with the presence or persistence of signs and symptoms in post-COVID-19 individuals [3]. The most common manifestations include pulmonary, musculoskeletal, hematologic, cardiovascular, endocrine, renal, and gastrointestinal symptoms [4]. Muscle weakness or fatigue, dyspnea, and sleep disturbance are among the most frequently reported symptoms [2].

Regarding musculoskeletal manifestations, in the acute phase of COVID-19 many subjects exhibit body composition changes, including a loss of muscle mass and strength. The muscular impairment can be attributed to various factors such as anorexia, malnutrition, and especially the severity of the illness. Hospitalized subjects have more significant pro-inflammatory states, oxidative stress, increased protein catabolism, and prolonged hospital stays, negatively impacting muscular mass. In addition, in subjects who require intensive care unit, invasive mechanical ventilation (IMV) and using neuromuscular blockers and corticosteroids negatively affect the peripheral muscles, the intercostal muscles, and the diaphragm, the primary muscle in charge of breathing [5–10].

Concerning the post-COVID phase, the pro-inflammatory state and endothelial dysfunction persist, as well as an increase in adipose tissue [11, 12]. Low muscle mass/strength and adipose tissue excess possess strongly interconnected physiopathologic mechanisms that exacerbate one another, resulting in a vicious cycle. This cycle leads to a reduction in protein synthesis, increased protein degradation, fat infiltration into skeletal muscle, promotion of lipotoxicity, exacerbation of inflammation [13], oxidative stress, and mitochondrial dysfunction [13–16]. Low muscle mass/strength loss and the accumulation of intramuscular fat contribute to muscle contractility impairment [17, 18].

The muscle strength can be easily assessed through handgrip strength (HGS). HGS has been demonstrated to be associated with whole-body muscle strength and skeletal muscle mass index (SMI), as well as pulmonary function, morbidity, and mortality in diverse populations [19–24].

The muscle quality index (MQI), obtained by dividing HGS by body mass index (BMI) (i.e., MQI = HGS/BMI), has emerged as a health and physical function indicator [25–28]. Existing evidence suggests that low MQI is linked to metabolic markers [28], metabolic syndrome [25], the prediction of cardiovascular disease risk factors [27], and physical function [28]. However, the association between the strength/BMI index and pulmonary function remains undefined. We aim to assess the association between MQI and pulmonary function in post-COVID-19 subjects.

## Methods

A cross-sectional study was conducted at the Instituto Nacional de Enfermedades Respiratorias “Ismael Cosío Villegas” in Mexico City.

The study focused on moderate to severe COVID-19 subjects with a confirmed diagnosis of COVID-19 by PCR testing. Moderate to severe COVID-19 was considered in those patients who, during the acute phase of the disease, required hospitalization with blood oxygen saturation ≤ 93%, PaO2/FiO2 ratio < 300 (arterial partial pressure of oxygen/fraction of inspired oxygen).

**Fig. 1:**
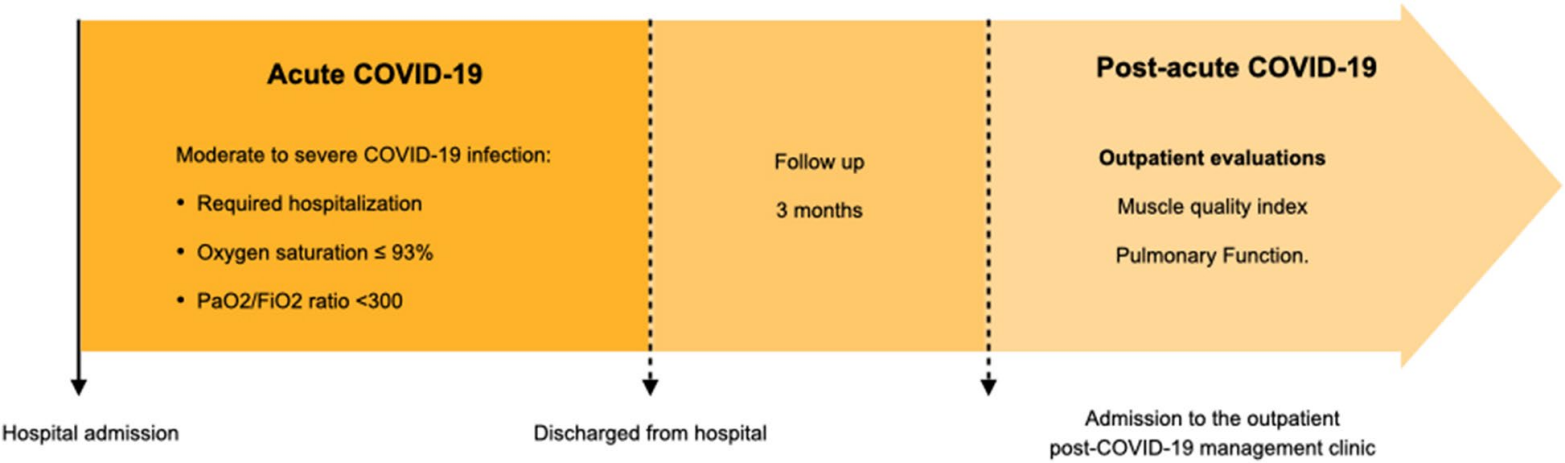
Study flow diagram

In the study, those patients during the acute phase were moderate to severe and were subsequently discharged were included. Data were collected from outpatient evaluations 3 months post-acute COVID-19 infection during routine clinical examinations of post-COVID-19 subjects between June 1, 2020, and May 30, 2023 (Fig. 1). Subjects who could not be contacted, declined to participate, or died before the follow-up visit were excluded.

### Outcome measures

Anthropometric, clinical, and demographic variables were evaluated during the post-COVID-19 clinical management delivered to patients at our institute.

#### Anthropometry

Weight and height were measured according to the manual reference for anthropometric standardization [29]. All subjects wore light clothing and were barefoot.

#### Body mass index (BMI)

BMI was estimated by weight in kilograms divided by height in meters squared.

#### Handgrip strength (HGS)

Handgrip strength was measured using a mechanical Smedley Hand Dynamometer (Stoelting, Wood Dale, UK) according to the technique described in Rodriguez et al., which consists of subjects standing with their arms stretched parallel to the trunk, then picking up the dynamometer and applying the maximum force with the dominant hand. The measurement was repeated three times, one minute apart, to avoid fatigue. The maximum value was recorded in kg [30].

#### Muscle quality index (MQI)

MQI was calculated by dividing HGS by BMI and subsequently categorised as follows: Low-MQI ≤ 50th percentile and High-MQI > 50th percentile, considering gender (50th percentile MQI: 0.54 for women and 0.99 for men).

#### Pulmonary function

Forced spirometry was performed using a portable spirometer (EasyOne Pro Lab, Ndd Medical Technologies Inc., Zürich, Switzerland) and carried out by an experienced respiratory medicine technician in accordance with American Thoracic Society/European Respiratory Society standards [31] The analyzed spirometric variables included forced expiratory volume in the first second (FEV1), forced vital capacity (FVC) before and after administering a bronchodilator, peak expiratory flow rate (PEFR), and maximum expiratory flow between 25 and 75% of the FVC (MEF 25–75). Following a 15-minute rest, participants performed a maximal forced inspiration and a forceful expiration using a nose clip. Spirometry reference values were derived from Mexican-American individuals [32].

#### Respiratory muscle strength

Maximal inspiratory pressures (MIP) and maximal expiratory pressures (MEP) were measured in accordance with ATS/ERS 2002 guidelines using MicroRPM equipment (CareFusion, Micromedical, UK) [33].

#### Carbon monoxide diffusing capacity (DLCO)

A skilled respiratory technician conducted tests for DLCO using EasyOne pro® equipment from Ndd Medical Technologies Inc., Zürich, Switzerland. The assessment accounted for altitude and hemoglobin, employing predicted values for the Latino population [34].

### Statistical analysis

Analyses were conducted using the commercially available software STATA version 14 (Stata Corp., College Station, TX, USA). Categorical variables were expressed as frequencies and percentages. The Shapiro-Wilk test assessed the normality of continuous variables; normal variables were expressed as mean and standard deviation, while non-normal variables were reported as median and percentiles 25–75. Comparisons between study groups (Low-MQI vs. High-MQI) were analyzed using the chi-square test for categorical variables and the t-test or Mann-Whitney U test for continuous variables. To evaluate the association between low-MQI and pulmonary function, linear regression models were performed using each variable of pulmonary function as a dependent variable and Low-MQI as an independent variable. The multivariate linear regression models were adjusted by bivariate analysis for variables with *p* < 0.10, such as age, diabetes, hypertension, ischaemic cardiopathy, and IMV, and multicollinearity was checked with the variance inflation factor. A *p*-value of 0.05 was considered statistically significant. A p-value of 0.05 was considered statistically significant.

## Results

Seven hundred and forty-eight patients were assessed, with a mean age of 54.61 ± 0.44 years; 63.90% were male and BMI were 30.39 ± 6.21. The low-MQI group comprised older individuals with a higher prevalence of hypertension, obesity and fatigue compared to the high-MQI group. Respect to hospitalary parameters during COVID-19 acute phase, low-MQI group had higher IMV, duration of IMV, and a longer hospital stay compared to the high-MQI group (Table 1).

**Table 1:** Demographic and clinical characteristics in Post-COVID-19 patients

	All *n* = 748	Low-MQI *n* = 375	High-MQI *n* = 373	p-value
Age, years	54.61 ± 0.44	58.01 ± 0.60	51.26 ± 0.61	< 0.001
Male, n (%)	478 (63.90)	238 (63.47)	240 (64.34)	0.803
BMI, kg/m^2^	30.39 ± 6.21	31.74 ± 7.25	29.04 ± 4.58	< 0.001
**Comorbidities**				
Diabetes, n(%)	260 (34.76)	142 (37.87)	118 (34.64)	0.074
Hypertension, n(%)	282 (37.70)	163 (43.47)	119 (31.90)	0.001
Obesity, n(%)	333 (44.52)	201 (53.60)	132 (35.39)	< 0.001
Ischemic cardiopathy, n(%)	54 (7.22)	33 (8.80)	21 (5.63)	0.094
Pulmonary disease, n(%)	119 (15.91)	65 (17.33)	54 (14.48)	0.286
Thyroid disease, n(%)	44 (5.88)	23 (6.13)	21 (5.63)	0.770
Hepatopathy, n(%)	19 (2.54)	12 (3.20)	7 (1.88)	0.250
HIV, n(%)	9 (1.20)	5 (1.33)	4 (1.07)	0.743
Asthma, n (%)	24 (3.21)	13 (3.49)	11 (2.93)	0.668
COPD, n (%)	13 (1.74)	9 (2.40)	4 (1.07)	0.165
**Post-COVID symptoms**				
Fatigue	40.11 (300)	44.53 (167)	35.66 (133)	0.013
Dyspnea	13.50 (101)	15.47 (58)	11.53 (43)	0.115
Anosmia	8.16 (61)	9.07 (34)	7.24 (27)	0.361
Muscular pain	37.30 (279)	38.67 (145)	35.9 (134)	0.438
**Hospitalary parameters**				
PaO2/FiO2	176.2 ± 91.2	170.20 ± 85.90	183.03 ± 96.69	0.180
Oxygen saturation, %	75.73 ± 16.09	73.87 ± 16.65	77.67 ± 15.29	0.012
VMI, n(%)	461 (61.96)	268 (71.66)	193 (52.16)	< 0.001
Duration VMI, d	16 [10–25]	18 [11–28]	15 [9–24]	0.043
Length of hospital stay, d	17 [10–29]	21 [12–35]	14 [9–23]	< 0.001

*HIV* Human immunodeficiency virus, *COPD* Chronic Obstructive Pulmonary Disease, *VMI* Ventilation Mechanical Invasive

In terms of pulmonary function, the Low-MQI group exhibited lower FEV1 in liters and percentage, FVC in liters and percentage, MEF 25–75, PEFR, FEV1/FVC ratio, DLCO, MIP, MEP than high-MQI group (Table 2).

**Table 2:** Pulmonary function according to muscle quality index

	All	Low MQI	High MQI	p-value
**Spirometry**				
**Pre Bronchodilator**				
FEV1, L	2.70 ± 0.02	2.50 ± 0.03	2.91 ± 0.03	< 0.001
FEV1, %	92.25 ± 0.66	90.15 ± 0.99	94.35 ± 0.86	< 0.001
FVC, L	3.34 ± 0.35	3.08 ± 0.04	3.60 ± 0.05	< 0.001
FVC, %	88.08 ± 18.14	85.94 ± 20.04	90.37 ± 15.76	< 0.001
MEF 25–75, %	3.18 ± 0.04	3.01 ± 0.06	3.36 ± 0.06	< 0.001
PEFR, L	8.74 ± 0.09	8.13 ± 0.13	9.35 ± 0.13	< 0.001
FEV1/FVC	2.75 ± 0.02	2.55 ± 0.03	2.96 ± 0.03	< 0.001
**Post Bronchodilator**				
FEV1, L	2.75 ± 0.02	2.55 ± 0.03	2.96 ± 0.03	< 0.001
FEV1, %	94.13 ± 18.02	92.12 ± 19.07	93.06 ± 16.6	< 0.001
FVC, L	3.33 ± 0.03	3.09 ± 0.04	3.58 ± 0.04	< 0.001
FVC, %	87.73 ± 0.68	85.80 ± 1.06	89.69 ± 0.84	0.004
MEF 25–75, %	3.49 ± 0.05	3.31 ± 0.07	3.68 ± 0.07	0.000
PEFR, L	8.93 ± 0.10	8.34 ± 0.14	9.52 ± 0.14	< 0.001
FEV1/FVC	0.83 ± 0.00	0.82 ± 0.00	0.83 ± 0.00	0.867
**Other pulmonary test**				
DLCO, %	72.20 ± 22.97	68.49 ± 23.46	75.98 ± 21.85	< 0.001
MIP, CmH_2_O	94.95 ± 1.03	89.75 ± 1.44	99.71 ± 1.41	< 0.001
MEP, CmH_2_O	117.85 ± 1.38	111.27 ± 1.84	124.44 ± 20.13	< 0.001

*FEV*1 Forced Expiratory Volume in One second, *FVC* Forced Vital Capacity, *MEF* Maximum Expiratory Flow between 25 and 75%, *PEFR* Peak Expiratory Flow Rate, *DLCO* Carbon monoxide diffusing capacity, *MIP* Maximal Inspiratory Pressures, *MEP* Maximal Expiratory Pressure

Additionally, Table 3 showed that Low-MQI was lower FEV1 in liters and percentage, FVC in liters and percentage, MEF 25–75, PEF, MIP, and MEP in both bivariate and multivariate models adjusted for age, diabetes, hypertension, ischaemic cardiopathy, and IMV. However, no significant difference was found in DLCO in either the crude or adjusted models.

**Table 3:** Association between low muscle quality index and pulmonary function

	Crude Model			Adjusted Model		
	β	CI (95%)	p-value	β	CI (95%)	p-value
**Pre Bronchodilator**						
FEV1, L	−0.40	−0.51 to − 0.30	< 0.001	− 0.25	− 0.34 to − 0.16	< 0.001
FEV1, %	−4.20	−6.80 to −1.60	0.002	−4.87	−7.58 to −2.17	< 0.001
FVC, L	− 0.52	− 0.65 to − 0.38	< 0.001	− 0.31	− 0.44 to − 0.19	< 0.001
FVC, %	− 4.42	− 7.19 to − 1.65	0.002	−3.53	− 6.45 to − 0.62	0.018
MEF 25–75, %	− 0.35	− 0.52 to − 0.17	< 0.001	− 0.25	− 0.42 to − 0.08	0.003
PEFR, L	−1.22	− 1.59 to − 0.85	< 0.001	− 0.79	−1.15 to − 0.43	< 0.001
FEV1/FVC	0.00	−0.00 to 0.01	0.900	−0.00	− 0.01 to 0.00	0.585
**Post Bronchodilator**						
FEV, L	−0.40	−0.51 to − 0.29	< 0.001	− 0.24	−0.34 to − 0.15	< 0.001
FEV1, %	−3.93	− 6.59 to −1.27	0.004	− 4.45	− 7.21 to − 1.68	0.002
FVC, L	−0.49	− 0.62 to − 0.36	< 0.001	−0.29	− 0.42 to − 0.17	< 0.001
FVC, %	−3.89	−6.56 to − 1.22	0.004	− 2.89	− 5.68 to − 0.11	0.041
MEF 25–75, %	−0.37	− 0.57 to − 0.17	< 0.001	−0.28	− 0.47 to − 0.09	0.003
PEFR, L	−1.17	− 1.57 to − 0.78	< 0.001	−0.74	−1.13 to − 0.35	< 0.001
FEV1/FVC	− 0.00	−0.00 to 0.00	0.868	−0.00	− 0.01 to 0.00	0.561
**Other pulmonary test**						
DLCO, %	26.64	−41.18 to 94.47	0.441	51.05	−20.21 to 122.32	0.160
MIP, CmH_2_O	−9.95	−13.94 to −5.97	< 0.001	− 5.84	− 9.83 to − 1.84	0.004
MEP, CmH_2_O	− 13.17	− 18.53 to − 7.80	< 0.001	−8.14	− 13.57 to − 2.71	0.003

*FEV*1 Forced Expiratory Volume in One second, *FVC* Forced Vital Capacity, *MEF* Maximum Expiratory Flow between 25 and 75%, *PEFR* Peak Expiratory Flow Rate, *DLCO* Maximum Diffusing Capacity of the Lung, *MIP* Maximal Inspiratory Pressures, *MEP* maximal Expiratory Pressure. Adjusted model by age, sex, diabetes, hypertension, ischaemic cardiopathy, and invasive mechanical ventilation

## Discussion

The primary finding of our research demonstrated a negative association between low-MQI and mechanical pulmonary function, as well as respiratory muscle strength in subjects with post-COVID syndrome.

In relation to pulmonary function, this is influenced by various factors such as age, sex, gestational weeks, muscular strength, the immune system, and exposure to toxic agents such as tobacco, wood smoke, asbestos, and respiratory infections. In a post-COVID-19 infection, a meta-analysis conducted by Lee and Cols demonstrated that impaired diffusion capacity was the most prevalent abnormality on pulmonary function tests at 35%. Restrictive patterns were identified in 8%, while persistent ground-glass opacities and pulmonary fibrosis had a prevalence of 34% [35]. FEV1 reduction is a significant predictor of mortality in the general population [36, 37], and serves as a marker for cardiovascular mortality [38]. It was observed that low-MQI independently predicts lower FEV1, as subjects with low-MQI had 4.87% less FEV1 (β: -4.87, CI 95%; − 7.58 to − 2.17, *p* < 0.001) and 3.53% less FVC (β: -3.53, CI 95%; − 6.45 to − 0.62, *p* < 0.018) than those with high-MQI, adjusted for confounding variables. Various researchers have demonstrated a negative association between low muscle mass, impaired in muscular performance and low muscle strength, or sarcopenia, with FEV1 and FVC [39–41]. van Gassel et al. observed that 3 months after hospital discharge in post-COVID-19 subjects requiring IVM, those subjects with decreased physical function had lower FEV1 and DLCO [42].

In the general population, both the BMI and central obesity have exhibited an inverse relationship with FEV1% and FVC% [43, 44].

Research indicates an inverse association between BMI and both FEV1 and FVC [6, 8, 45]. This is attributed to the detrimental impact of excess adiposity, particularly centralized fat, on pulmonary function. Studies by Kwack and Cols demonstrated significant associations between subcutaneous thoracic fat, intra-thoracic fat, subcutaneous abdominal fat, and lower FEV1 and FVC [46]. The presence of excessive adipose tissue, particularly around the chest and abdomen, has been linked to worse lung function, likely due to difficulties in respiratory mechanics. The latter results from restrictions on lung expansion and increased resistance to diaphragmatic contraction during respiration, consequently causing reduced lung volume [45, 46].

Nonetheless, Koo et al. demonstrated that COPD patients with sarcopenic obesity exhibited poorer lung function compared to individuals without sarcopenia or obesity [47]. Moreover, those with sarcopenic obesity presented elevated levels of C-reactive protein, IL-6, and reduced exercise tolerance [48]. Additionally, sarcopenic obesity is linked to an increased risk of restrictive lung disease among the elderly [49].

As previously stated, a series of interconnected mechanisms between low muscle mass and strength and excess adipose tissue lead to alterations in the catabolism and anabolism of proteins and glycogen, as well as energy utilization [13, 50]. Additionally, mitochondrial dysfunction, diminishing muscle fiber number, decreased capillary density, increased oxidative stress, and inflammation occur along with adipose tissue infiltration. Such events promote myofibril atrophy and loss of muscle function both in peripheral muscles and those responsible for respiration, such as the diaphragm and intercostal muscles [13–16, 51].

Low muscle mass/strength and an excess of adipose tissue independently impair lung function [24, 40, 44]. Moreover, they possess synergistic mechanisms that result in a vicious cycle [47, 48].

The MQI is an emerging indicator of health and physical function [28] that represents a valuable tool for clinical practice, as it is a low-cost and easy tool to assess skeletal muscle quality, taking into account the important relationship between muscle strength and adipose tissue, which and predicting lung function, respiratory muscle strength in post-COVID-19 subjects. The assessment of MQI allows the identification of subjects at risk for opportune therapeutic management.

In terms of respiratory muscle functionality, in post-COVID-19 subjects who were hospitalized for severe COVID-19 infection, after resolution of the active infection, there was a lower thickening ratio between diaphragm thickness at end-inspiration/end-expiration compared with non-COVID subjects [52]. Inspiratory muscle functions can be assessed by MIP, while expiratory muscle strength is evaluated through MEP or PEFR.

Our study demonstrated a negative association between low-MQI and respiratory muscle strength, as evaluated by PEF, MIP, and MEP. Participants with low-MQI exhibited a 0.74 L lower PEFR than those with High-MQI (β: -0.79, CI 95%; − 1.15 to − 0.43, *p* < 0.001). PEFR is associated with respiratory muscle strength, low skeletal muscle mass, and sarcopenia [21, 53, 54] Kera and Cols. employed PEFR to define respiratory sarcopenia [54]. The PEFR was found to be associated with the 5-year mortality rate in an older population [55]. Additionally, we observed that individuals with low-MQI exhibited 8.14 cmH2O lower MEP (β: - 8.14, CI 95%; − 13.57 to − 2.71, *p* = 0.003) and 5.84 cmH2O less MIP (β: - 5.84, CI 95%; − 9.83 to − 1.84, *p* = 0.004) than those with High-MQI. MEP evaluates the strength of abdominal and intercostal muscles, while MIP measures the diaphragm’s strength—the most crucial muscle for respiration [56] Various studies have demonstrated a positive association between HGS and respiratory muscle strength; Shin and Cols showed that in adults over 60 years of age, for each kilogram of HGS, MIP increased by 1.96 cmH2O and MEP by 1.10 cmH2O [56]. Moreover, it has been noted that protein synthesis deterioration and mitochondrial degradation are more prominent in sarcopenic obesity than in sarcopenia or obesity alone [51].

### Strengths and limitations

This study possesses inherent limitations due to its cross-sectional design, such as not being able to determine causality between variables. In addition, we do not know the subjects’ lung function and muscle quality before COVID-19 infection. Another significant limitation is that as this is the first study to evaluate the association between MQI and lung function in the post-COVID syndrome population, it is impossible to contrast our results in other post-COVID syndrome populations. Nonetheless, the study’s strengths include a large sample size to provide sufficient statistical power for conducting multiple linear regression analyses and adjusting for confounding variables.

## Conclusions

The low-MQI serves as an independent predictor linked to pulmonary function parameters among individuals experiencing post-COVID-19 syndrome. The MQI could function as an indicator that determines the requirement for muscle training within pulmonary rehabilitation programs.

## Data Availability

The datasets generated and/or analysed during the current study are not publicly available due to the fact that individual privacy could be compromised but are available from the corresponding author on reasonable request.

## Abbreviations

BMI: Body mass index
FEV1: Forced expiratory volume in 1 second
FVC: Forced vital capacity
DLCO: Carbon monoxide diffusing capacity
HSG: Handgrip strength
MEP: Maximal expiratory pressure
MEF 25–75: Maximum expiratory flow between 25 and 75%
MIP: Maximal inspiratory pressures
MQI: Muscle quality index
SARS-CoV-2: Severe acute respiratory syndrome coronavirus-2
SMI: Skeletal muscle mass index

## References

[1] Shah W, Hillman T, Playford ED, Hishmeh L (2021). Managing the long term effects of covid-19: summary of NICE, SIGN, and RCGP rapid guideline. Bmj..

[2] Huang L, Yao Q, Gu X, Wang Q, Ren L, Wang Y (2021). 1-year outcomes in hospital survivors with COVID-19: a longitudinal cohort study. Lancet..

[3] Fernández-de-Las-Peñas C, Palacios-Ceña D, Gómez-Mayordomo V, Florencio LL, Cuadrado ML, Plaza-Manzano G (2021). Prevalence of post-COVID-19 symptoms in hospitalized and non-hospitalized COVID-19 survivors: a systematic review and meta-analysis. Eur J Intern Med.

[4] Gupta A, Madhavan MV, Sehgal K, Nair N, Mahajan S, Sehrawat TS (2020). Extrapulmonary manifestations of COVID-19. Nat Med.

[5] Reid MB, Li YP (2001). Tumor necrosis factor-alpha and muscle wasting: a cellular perspective. Respir Res.

[6] Yang T, Li Z, Jiang L, Xi X (2018). Corticosteroid use and intensive care unit-acquired weakness: a systematic review and meta-analysis. Critical care (London, England).

[7] Forcina L, Miano C, Scicchitano BM, Rizzuto E, Berardinelli MG, De Benedetti F (2019). Increased circulating levels of Interleukin-6 affect the redox balance in skeletal muscle. Oxidative Med Cell Longev.

[8] Disser NP, De Micheli AJ, Schonk MM, Konnaris MA, Piacentini AN, Edon DL (2020). Musculoskeletal consequences of COVID-19. J Bone Joint Surg Am.

[9] Pacifico J, Geerlings MAJ, Reijnierse EM, Phassouliotis C, Lim WK, Maier AB (2020). Prevalence of sarcopenia as a comorbid disease: a systematic review and meta-analysis. Exp Gerontol.

[10] González-Islas D, Sánchez-Moreno C, Orea-Tejeda A, Hernández-López S, Salgado-Fernández F, Keirns-Davis C (2022). Body composition and risk factors associated with sarcopenia in post-COVID patients after moderate or severe COVID-19 infections. BMC Pulm Med..

[11] Lemos MM, Cavalini GR, Pugliese Henrique CR, Perli VAS, de Moraes MG, Marchiori LLM (2022). Body composition and cardiorespiratory fitness in overweight or obese people post COVID-19: a comparative study. Front Physiol.

[12] Jacobs LMC, Wintjens M, Nagy M, Willems L, Ten Cate H, Spronk HMH (2023). Biomarkers of sustained systemic inflammation and microvascular dysfunction associated with post-COVID-19 condition symptoms at 24 months after SARS-CoV-2-infection. Front Immunol.

[13] Kalinkovich A, Livshits G (2017). Sarcopenic obesity or obese sarcopenia: a cross talk between age-associated adipose tissue and skeletal muscle inflammation as a main mechanism of the pathogenesis. Ageing Res Rev.

[14] Aon MA, Bhatt N, Cortassa SC (2014). Mitochondrial and cellular mechanisms for managing lipid excess. Front Physiol.

[15] Di Meo S, Iossa S, Venditti P (2017). Skeletal muscle insulin resistance: role of mitochondria and other ROS sources. J Endocrinol.

[16] Wu H, Ballantyne CM (2017). Skeletal muscle inflammation and insulin resistance in obesity. J Clin Invest.

[17] Baker JF, Mostoufi-Moab S, Long J, Zemel B, Ibrahim S, Taratuta E (2018). Intramuscular fat accumulation and associations with body composition, strength, and physical functioning in patients with rheumatoid arthritis. Arthritis care & research.

[18] Wu H, Liu M, Chi VTQ, Wang J, Zhang Q, Liu L (2019). Handgrip strength is inversely associated with metabolic syndrome and its separate components in middle aged and older adults: a large-scale population-based study. Metab Clin Exp.

[19] Rantanen T, Harris T, Leveille SG, Visser M, Foley D, Masaki K (2000). Muscle strength and body mass index as long-term predictors of mortality in initially healthy men. J Gerontol A Biol Sci Med Sci.

[20] Izawa KP, Watanabe S, Osada N, Kasahara Y, Yokoyama H, Hiraki K (2009). Handgrip strength as a predictor of prognosis in Japanese patients with congestive heart failure. Eur J Cardiovasc Prev Rehabil.

[21] Ro HJ, Kim DK, Lee SY, Seo KM, Kang SH, Suh HC (2015). Relationship between respiratory muscle strength and conventional Sarcopenic indices in young adults: a preliminary study. Ann Rehabil Med.

[22] Sayer AA, Kirkwood TB (2015). Grip strength and mortality: a biomarker of ageing?. Lancet..

[23] Liu X, Li P, Wang Z, Lu Y, Li N, Xiao L (2019). Evaluation of isokinetic muscle strength of upper limb and the relationship with pulmonary function and respiratory muscle strength in stable COPD patients. Int J Chron Obstruct Pulmon Dis.

[24] Martínez-Luna N, Orea-Tejeda A, González-Islas D, Flores-Cisneros L, Keirns-Davis C, Sánchez-Santillán R (2022). Association between body composition, sarcopenia and pulmonary function in chronic obstructive pulmonary disease. BMC Pulm Med..

[25] Moreira MA, Vafaei A, da Câmara SMA, Nascimento RAD, de Morais M, Almeida MDG (2020). Metabolic syndrome (MetS) and associated factors in middle-aged women: a cross-sectional study in Northeast Brazil. Women Health.

[26] Choi S, Nah S, Jang H, Moon J, Han S. Association between relative handgrip strength and chronic lower Back pain: a Nationwide cross-sectional analysis of the Korea National Health and nutrition examination survey. Int J Environ Res Public Health. 2021;18(20) 10.3390/ijerph182010770 PMC8535507 34682530

[27] Gao Y, Huang H, Ni C, Feng Y, Yu J, Huang Y (2022). Comparison of five expressions of handgrip strength for predicting cardiovascular disease risk factors in Chinese middle-aged community residents. Front Public Health.

[28] Caamaño-Navarrete F, Jerez-Mayorga D, Alvarez C, Del-Cuerpo I, Cresp-Barría M, Delgado-Floody P. Muscle quality index in morbidly obesity patients related to metabolic syndrome markers and cardiorespiratory fitness. Nutrients. 2023;15(11) 10.3390/nu15112458 PMC10254905 37299421

[29] Tg L. Anthropometric standardization reference manual. Human kinetics books. 1988:55–68.

[30] Rodríguez-García WD, García-Castañeda L, Orea-Tejeda A, Mendoza-Núñez V, González-Islas DG, Santillán-Díaz C (2017). Handgrip strength: reference values and its relationship with bioimpedance and anthropometric variables. Clinical nutrition ESPEN.

[31] Graham BL, Steenbruggen I, Miller MR, Barjaktarevic IZ, Cooper BG, Hall GL (2019). Standardization of Spirometry 2019 update. An official American Thoracic Society and European Respiratory Society technical statement. Am J Respir Crit Care Med.

[32] Hankinson JL, Odencrantz JR, Fedan KB (1999). Spirometric reference values from a sample of the general U.S. population. Am J Respir Crit Care Med.

[33] ATS/ERS Statement on respiratory muscle testing (2002). Am J Respir Crit Care Med.

[34] Vázquez-García JC, Pérez-Padilla R, Casas A, Schönffeldt-Guerrero P, Pereira J, Vargas-Domínguez C (2016). Reference values for the diffusing capacity determined by the single-breath technique at different altitudes: the Latin American single-breath diffusing capacity reference project. Respir Care.

[35] Lee JH, Yim JJ, Park J (2022). Pulmonary function and chest computed tomography abnormalities 6-12 months after recovery from COVID-19: a systematic review and meta-analysis. Respir Res.

[36] Schünemann HJ, Dorn J, Grant BJ, Winkelstein W, Trevisan M (2000). Pulmonary function is a long-term predictor of mortality in the general population: 29-year follow-up of the Buffalo health study. Chest..

[37] Ji Z, de Miguel-Diez J, Castro-Riera CR, Bellon-Cano JM, Gallo-Gonzalez V, Giron-Matute WI, et al. Differences in the outcome of patients with COPD according to body mass index. J Clin Med. 2020;9(3) 10.3390/jcm9030710 PMC7141195 32151054

[38] Sin DD, Man SP (2003). Why are patients with chronic obstructive pulmonary disease at increased risk of cardiovascular diseases? The potential role of systemic inflammation in chronic obstructive pulmonary disease. Circulation..

[39] Moon JH, Kong MH, Kim HJ (2015). Implication of sarcopenia and Sarcopenic obesity on lung function in healthy elderly: using Korean National Health and nutrition examination survey. J Korean Med Sci.

[40] Chen L, Liu X, Wang Q, Jia L, Song K, Nie S (2020). Better pulmonary function is associated with greater handgrip strength in a healthy Chinese Han population. BMC Pulm Med.

[41] Benz E, Wijnant SRA, Trajanoska K, Arinze JT, de Roos EW, de Ridder M, et al. Sarcopenia, systemic immune-inflammation index and all-cause mortality in middle-aged and older people with COPD and asthma: a population-based study. ERJ open research. 2022;8(1) 10.1183/23120541.00628-2021 PMC8752940 35036418

[42] van Gassel RJJ, Bels J, Remij L, van Bussel BCT, Posthuma R, Gietema HA (2021). Functional outcomes and their association with physical performance in mechanically ventilated coronavirus disease 2019 survivors at 3 months following hospital discharge: a cohort study. Crit Care Med.

[43] Ochs-Balcom HM, Grant BJ, Muti P, Sempos CT, Freudenheim JL, Trevisan M (2006). Pulmonary function and abdominal adiposity in the general population. Chest..

[44] Pan J, Xu L, Lam TH, Jiang CQ, Zhang WS, Jin YL (2017). Association of adiposity with pulmonary function in older Chinese: Guangzhou biobank cohort study. Respir Med.

[45] Ohara DG, Moreira YP, Silva CFR, Matos AP, Jamami M, Gama TO (2022). Impaired pulmonary function is associated with dynapenia, but not with abdominal obesity and dynapenic abdominal obesity in older adults. European geriatric medicine.

[46] Kwack WG, Kang YS, Jeong YJ, Oh JY, Cha YK, Kim JS (2019). Association between thoracic fat measured using computed tomography and lung function in a population without respiratory diseases. Journal of thoracic disease.

[47] Koo HK, Park JH, Park HK, Jung H, Lee SS (2014). Conflicting role of sarcopenia and obesity in male patients with chronic obstructive pulmonary disease: Korean National Health and nutrition examination survey. PLoS One.

[48] Joppa P, Tkacova R, Franssen FM, Hanson C, Rennard SI, Silverman EK (2016). Sarcopenic obesity, functional outcomes, and systemic inflammation in patients with chronic obstructive pulmonary disease. J Am Med Dir Assoc.

[49] Lee SE, Park JH, Kim KA, Kang YS, Choi HS (2020). Association between Sarcopenic obesity and pulmonary function in Korean elderly: results from the Korean National Health and nutrition examination survey. Calcif Tissue Int.

[50] Hunter GR, Singh H, Carter SJ, Bryan DR, Fisher G (2019). Sarcopenia and its implications for metabolic health. J Obes.

[51] Kim KW, Baek MO, Yoon MS, Son KH (2021). Deterioration of mitochondrial function in the human intercostal muscles differs among individuals with sarcopenia, obesity, and sarcopenic obesity. Clin Nutr.

[52] Farr E, Wolfe AR, Deshmukh S, Rydberg L, Soriano R, Walter JM (2021). Diaphragm dysfunction in severe COVID-19 as determined by neuromuscular ultrasound. Ann Clin Transl Neurol.

[53] Kera T, Kawai H, Hirano H, Kojima M, Fujiwara Y, Ihara K (2018). Relationships among peak expiratory flow rate, body composition, physical function, and sarcopenia in community-dwelling older adults. Aging Clin Exp Res.

[54] Kera T, Kawai H, Hirano H, Kojima M, Watanabe Y, Motokawa K (2019). Definition of respiratory sarcopenia with peak expiratory flow rate. J Am Med Dir Assoc.

[55] Cook NR, Evans DA, Scherr PA, Speizer FE, Taylor JO, Hennekens CH (1991). Peak expiratory flow rate and 5-year mortality in an elderly population. Am J Epidemiol.

[56] Shin HI, Kim DK, Seo KM, Kang SH, Lee SY, Son S (2017). Relation between respiratory muscle strength and skeletal muscle mass and hand grip strength in the healthy elderly. Ann Rehabil Med.

